# An Overview of Contrasting Experimental Results on Dynamics of Kinesin-1 Molecular Motors: Insight into the Underlying Mechanism

**DOI:** 10.3390/biom15101453

**Published:** 2025-10-14

**Authors:** Ping Xie

**Affiliations:** Laboratory of Soft Matter Physics, Institute of Physics, Chinese Academy of Sciences, Beijing 100190, China; pxie@aphy.iphy.ac.cn

**Keywords:** kinesin, dynamics, controversial experimental results, mechanochemistry

## Abstract

The conventional kinesin (kinesin-1) molecular motor is a prototypical member of the kinesin superfamily. It can processively step on microtubules toward the plus end by hydrolyzing ATP molecules, performing the biological function of shuttling cargos in cells. Its dynamics have been thoroughly studied using various methods including biochemical measurement, single molecule imaging, single molecule optical trapping, and so on. While most of the experiments yielded consistent results on the dynamics of the motor, a lot of conflicting experimental results have also been presented. Here, a brief review is given of the diverse conflicting experimental results. Furthermore, a model for the chemomechanical coupling of the motor is briefly reviewed, which can consistently and quantitatively explain these conflicting experimental results in addition to the other experimental results. A consistent explanation of the diverse conflicting experimental results with the same model is an essential criterion for determining the correctness of the model.

## 1. Introduction

Kinesin proteins constitute a large superfamily of molecular motors, which can be categorized into 14 families and an uncategorized family called the ‘orphan kinesin’ [1,2,3,4,5]. Kinesin-1 was the first-found member of the superfamily [6,7] and has been thoroughly studied [8,9,10,11,12]. In this review, unless otherwise pointed out, we concentrate on the wild type (WT) kinesin-1 motor, which is simply called the ‘kinesin motor.’ The kinesin motor has a homodimeric form consisting of a pair of identical motor domains (also called ‘heads’) that are linked together by a long coiled-coil stalk through their two flexible neck linkers (NLs), with each NL having 14 amino acids [13]. After landing on microtubule (MT), powered by ATP binding and hydrolysis, the kinesin motor can step along MT toward the plus end with a velocity of about 800 nm/s for a long distance of about 1 μm before dissociation [14,15], performing the biological function of shuttling cargos in cells [1,2,3,4,5]. An interesting and important issue for the kinesin motor is how the chemical energy arising from the ATP binding and hydrolysis is converted to the mechanical energy of the directional motion (i.e., the mechanism of chemomechanical coupling). To address the issue, in addition to structural studies [13,16,17,18,19,20,21], the dynamics of the motor have also been investigated in great detail by using various methods, including biochemical measurement [22,23,24,25], single molecule imaging [26,27,28,29], single molecule optical trapping [14,15,30,31,32,33,34,35,36,37,38,39], coarse-grained and atomistic molecular dynamics (AMD) simulation [40,41,42,43,44,45,46,47], theoretical modeling and analysis [48,49,50,51,52,53,54,55,56,57,58], etc.

It was definitively determined that the dimeric kinesin motor processively steps along an MT filament in a hand-over-hand way and rarely takes sideways steps [59,60]. It takes either forward steps or backward steps with the size of about 8 nm that is equal to the period of tubulins on an MT filament [28,29,30,39]. Under no load and a small backward (minus-end-directed) load, the motor mainly steps forward and occasionally steps backward [14,15,30,31,32,33,34,35,36,37,38,39]. As the backward load increases, the number of the forward steps decreases while that of the backward steps increases. Under a backward load of about 6–8 pN—the stall force—the motor steps forward and backward with the same probability, with no net movement [31,32,33,34,35,36,37,38]. Under a backward load larger than the stall force, the motor mainly steps backward [36,61,62]. Both the forward and backward steps require ATP binding and hydrolysis [30,34,36,61].

However, it is perplexing that a plethora of contrasting experimental results have been presented on the dynamics of the kinesin motor. Here, we present a brief review on the contrasting experimental results. Furthermore, we briefly review a model for the chemomechanical coupling of the kinesin, which can consistently explain these contrasting experimental results as well as other experimental results. A consistent explanation of the diverse contrasting experimental data using the same model is crucial to the mechanism of the chemomechanical coupling of the kinesin.

## 2. ATP Binding Occurs in One-Head-Bound or Two-Heads-Bound State

It is known that during the processive motion, the kinesin dimer alternates between one-head-bound (1HB, with one head bound to MT) and two-heads-bound (2HB, with both heads bound to MT) states [26,27,28,29,39,63,64,65]. The directional motion is powered by ATP binding and hydrolysis [30,34,36,61]. Thus, an interesting issue is whether ATP binding occurs in the 1HB or 2HB state. Three independent experiments have been performed to address the issue [26,27,28].

**Figure 1:**
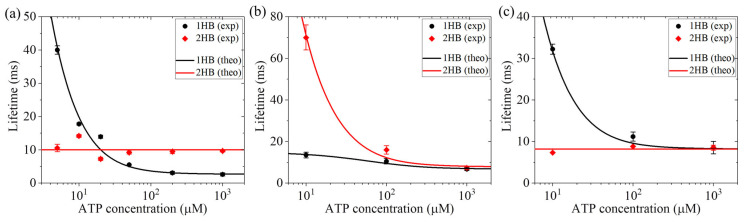
Lifetimes of 1HB and 2HB states of kinesin motor in a stepping cycle versus ATP concentration. (**a**) The experimental results (symbols) are from Isojima et al. [27]. The theoretical results (lines) are from Refs. [66,67]. (**b**) The experimental results (symbols) are from Mickolajczyk et al. [26]. The theoretical results (lines) are from Ref. [66]. (**c**) The experimental results (symbols) are from Wolff et al. [28]. The theoretical results (lines) are from Ref. [66].

In one experiment, Isojima et al. [27] attached a gold particle (20 nm or 40 nm) to a cysteine amino acid (S55C) at the rear position of one head of the human kinesin dimer via two polypeptide chains. They imaged the particle using the total internal reflection dark-field microscopy to measure the lifetimes of the 1HB and 2HB states of the dimer in a stepping cycle versus ATP concentration. They found that as the ATP concentration decreases, the lifetime of the 1HB state increases largely, whereas that of the 2HB state remains unchanged (Figure 1a). This indicates that ATP binding occurs in the 1HB state.

In another experiment, Mickolajczyk et al. [26] attached a 30 nm gold particle to the center right of one head of the *Drosophila* kinesin dimer via a short linker of the contour length of only 2.91 nm. They imaged the particle using the interferometric scattering microscopy to measure the lifetimes of the 1HB and 2HB states of the dimer in a stepping cycle versus ATP concentration. They found that as the ATP concentration decreases, the lifetime of the 1HB state increases slightly, whereas that of the 2HB state increases largely (Figure 1b), which is contrary to that observed by Isojima et al. [27]. The experimental results measured by Mickolajczyk et al. [26] indicate that ATP binding occurs mainly in the 2HB state.

In the third experiment, Wolff et al. [28] labeled an approximately 1 nm–sized fluorophore to a solvent-exposed cysteine located at the C-terminal end of the α6 helix on one head. They employed the interferometric MINFLUX microscope to measure the lifetimes of the 1HB and 2HB states of the dimer in a stepping cycle versus ATP concentration. They found that as the ATP concentration decreases, the lifetime of the 1HB state increases largely, whereas that of the 2HB state remains constant (Figure 1c), which is similar to that observed by Isojima et al. [27].

Taken together, the experimental results by Isojima et al. [27] and those by Wolff et al. [28] indicated that ATP binding occurs in the 1HB state. On the contrary, the experimental results by Mickolajczyk et al. [26] indicated that ATP binding occurs mainly in the 2HB state.

## 3. Velocity Versus Backward Load Has a Sigmoid or Linear Form

As the kinesin motor performs the biological function of shuttling cargo, it is constructive to determine the dependence of the motor’s velocity upon the load acting on the motor. The load dependence of the velocity was usually determined using the single molecule optical trapping techniques, where a large-sized bead is attached to the motor’s stalk. Two types of the trap have been utilized in the experiments. One type is termed as the ‘fixed trap,’ where the trap is kept stationary during the processive motion of the motor [15,68]. For the fixed trap, the load on the motor changes as the motor processively moves. The other type is termed as the ‘movable trap,’ which can be achieved by controlling the position of the trap utilizing acoustic optical deflectors during the processive motion of the motor to ensure a constant load on the motor as the motor processively moves [69]. Using both types of the trap, the dependence of the motor’s velocity on the load has been extensively studied [32,33,34,35,36,37,38,39,70,71,72,73]. In this section, we briefly review the contrasting experimental results about the velocity versus backward load. Throughout, the backward (forward) load is defined as having the positive (negative) value.

**Figure 2:**
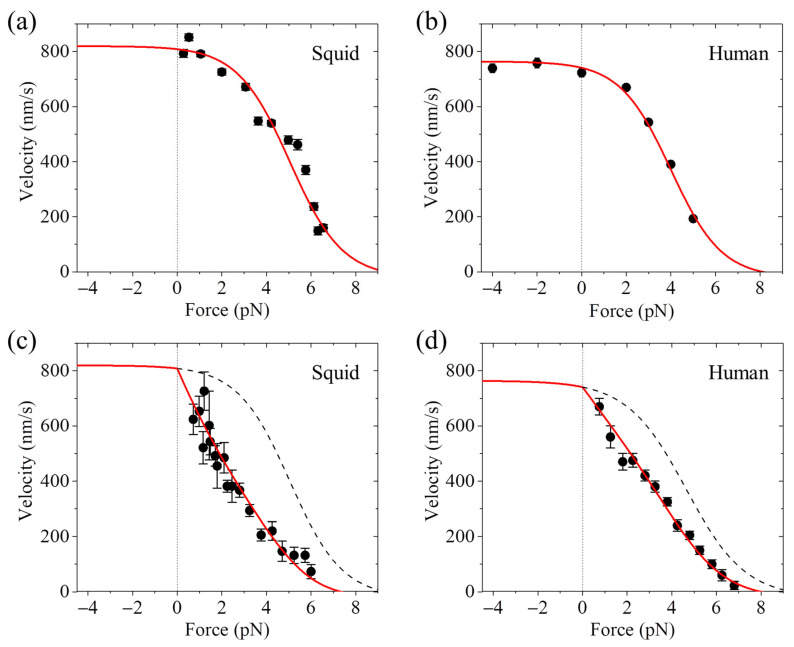
Force–velocity relation for squid optic lobe and human kinesin motors. The backward (forward) force is defined as having the positive (negative) value. (**a**) Results for the squid optic lobe kinesin measured with the *movable* trap. The experimental results (symbols) are from Visscher et al. [33]. (**b**) Results for the human kinesin measured with the *movable* trap. The experimental results (symbols) are from Andreasson et al. [37]. (**c**) Results for the squid optic lobe kinesin measured with the *fixed* trap. The experimental results (symbols) are from Svoboda and Block [72]. (**d**) Results for the human kinesin motor measured with the *fixed* trap. The experimental results (symbols) are from Kaseda et al. [71]. In (**a**) through (**d**), the theoretical results (lines) are from Ref. [74]. The theoretical results under the forward load correspond to Case II with the motor being on the upper side of MT and the bottom side of MT being fixed to the stage surface. For comparison, in (**c**) and (**d**), the corresponding theoretical results for the *movable* trap are shown by dashed lines.

Using the movable trap, it was generally obtained that the motor’s velocity decreases slowly with the increase in the backward load when the load is small, decreases quickly when the load has the medium and large values, and decreases slowly again when the load is near the stall force [33,35,37,70]; namely, the relation between the velocity and backward load exhibits a sigmoid form (Figure 2a,b). In contrast, using the fixed trap, it was generally obtained that the velocity decreases almost linearly with the increase in the backward load when the load is smaller than the stall force [32,34,39,71,72,73]; namely, the relation between the velocity and backward load exhibits a nearly linear form (Figure 2c,d). In particular, the same research group of Block et al. [33,72] evidently showed that the same squid optic lobe kinesin has the sigmoid form of the velocity versus backward load determined using the movable trap (Figure 2a), whereas it has the nearly linear form determined using the fixed trap (Figure 2c). For the same human kinesin, using the movable trap, Andreasson et al. [37] determined the sigmoid form of the velocity versus the backward load (Figure 2b), whereas using the fixed trap, Kaseda et al. [71] determined the nearly linear form (Figure 2d).

In short, using the movable optical trap, it was determined that the relation between the velocity and backward load generally has a sigmoid form [33,35,37,70], as shown in Figure 2a,b. In contrast, using the fixed optical trap, it was determined that the relation between the velocity and backward load generally has a nearly linear form [32,34,39,71,72,73], as shown in Figure 2c,d.

## 4. Velocity Is Independent of or Decreases with or Increases with Forward Load

In this section, we briefly review the contrasting single molecule optical trapping results on the relation between the velocity and forward load.

**Figure 3:**
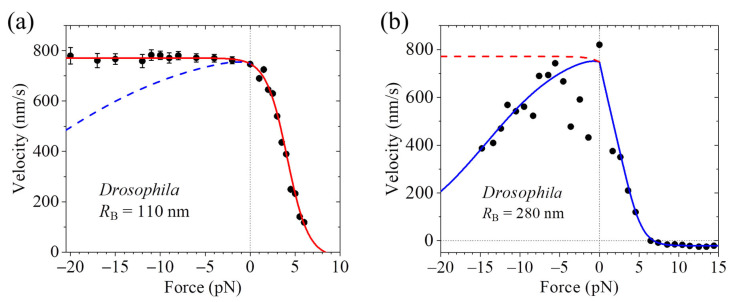
Force–velocity relation for *Drosophila* kinesin motor. The backward (forward) force is defined as having the positive (negative) value. (**a**) The experimental results (symbols) are from Andreasson et al. measured using the movable trap [37]. (**b**) The experimental results (symbols) are from Carter and Cross measured using the fixed trap [36]. In (**a**) and (**b**), the theoretical results (lines) are from Ref. [74]. Blue solid and dashed lines represent the theoretical results for Case I with the motor being on the left side of MT (viewed toward the plus end) and the bottom side of MT being fixed to the stage surface. Red solid and dashed lines represent the theoretical results for Case II with the motor being on the upper side of MT.

Some optical trapping assays obtained that the motor’s velocity is nearly independent on the forward load [37]. On the contrary, other optical trapping assays obtained that as the magnitude of the forward load increases, the velocity decreases slowly when the magnitude of the forward load is small and decreases quickly when the magnitude of the forward load is large [36,70]. In particular, for the same *Drosophila* kinesin, the assays by Andreasson et al. [37] obtained that the velocity is nearly independent on the forward load (Figure 3a), and by contrast, the assays by Carter and Cross [36] obtained that the velocity decreases as the magnitude of the forward load increases (Figure 3b).

**Figure 4:**
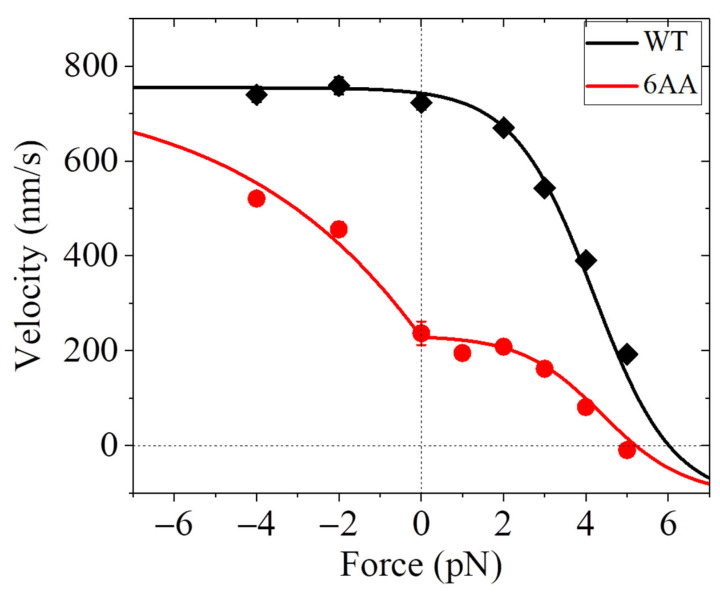
Force–velocity relation for WT and mutant human kinesin motors. The backward (forward) force is defined as having the positive (negative) value. The WT human kinesin is represented by WT while the mutant one is represented by 6AA with each of the two NLs being extended by adding six amino acids. The experimental results (symbols) are from Andreasson et al. measured using the movable trap [37]. The theoretical results (lines) are from Ref. [77], which were calculated with the WT and 6AA having the same parameter values except that under no load *P*_0_ = 1 for the WT and *P*_0_ = 0.37 for the 6AA. The theoretical results under the forward load correspond to Case II with the motor being on the upper side of MT and the bottom side of MT being fixed to the stage surface.

**Figure 5:**
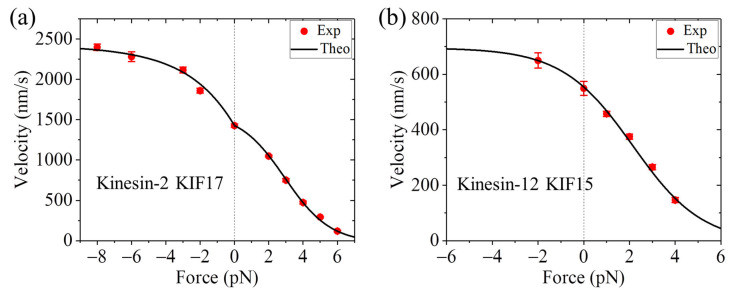
Force–velocity relation for kinesin-2 KIF17 and kinesin-12 KIF15 motors. The backward (forward) force is defined as having the positive (negative) value. (**a**) Results for the kinesin-2 KIF17. The experimental results (symbols) are from Milic et al. measured using the movable trap [75]. (**b**) Results for the kinesin-12 KIF15. The experimental results (symbols) are from Milic et al. measured using the movable trap [76]. In (**a**) and (**b**), the theoretical results (lines) are from Ref. [78]. The theoretical results under the forward load correspond to Case II with the motor being on the upper side of MT and the bottom side of MT being fixed to the stage surface.

Additionally, it is noted that some optical trapping assays obtained that while for the human kinesin motor, the velocity is nearly independent on the forward load, for the corresponding mutant motor with the NL in each head being extended by adding six additional amino acids, the velocity increases as the magnitude of the forward load increases [37] (Figure 4). Interestingly, some optical trapping assays obtained that for some WT members of other kinesin families, such as KIF17 dimer of kinesin-2 family and KIF15 dimer of kinesin-12 family, the velocity also increases as the magnitude of the forward load increases [75,76] (Figure 5).

In summary, some optical trapping assays obtained that the velocity of the WT kinesin motor is nearly independent on the forward load [37], as shown in Figure 3a, whereas others obtained that the velocity of the WT kinesin motor decreases as the magnitude of the forward load increases [36,70], as shown in Figure 3b. The optical trapping assays obtained that while for the WT human kinesin motor, the velocity is nearly independent on the forward load, for the corresponding mutant motor with the NL extension, the velocity increases as the magnitude of the forward load increases [37], as shown in Figure 4. The optical trapping assays obtained that for some WT members of other kinesin families, such as KIF17 dimer of kinesin-2 family and KIF15 dimer of kinesin-12 family, the velocity also increases as the magnitude of the forward load increases [75,76], as shown in Figure 5.

## 5. Kinesin Pauses for a Short or Long Time upon Reaching Roadblocks

In cells, during the processive motion of a kinesin motor on MT, other MT-associated proteins can often bind to the MT, which act as roadblocks affecting the dynamics of the kinesin motor. Consequently, a lot of in vitro experimental studies have been performed on the dynamics of the kinesin motors with the presence of stationary roadblocks on MTs [79,80,81,82,83,84,85].

Most experimental results indicated that upon reaching the roadblock of a small size (e.g., the rigor-binding mutant of kinesin), the kinesin motor dissociates from MT after pausing for a short time of a few tens or hundreds of milliseconds [79,80,81], which is evidently shorter than the residence time of about 1 s on MT during the processive stepping in the absence of the roadblock [37]. The experimental results indicated that upon reaching the boundary of the tau cohesive islands, the kinesin motor also dissociates from MT rapidly, similar to reaching the small roadblock [86]. On the contrary, some experimental results indicated that upon reaching the roadblock of a large size (e.g., the 20 nm quantum dot, avidin, etc.), the kinesin motor can pause for a long time before dissociation, which is evidently longer than the residence time of about 1 s on MT in the absence of the roadblock [83,84,85].

Collectively, the experimental results indicated that different-sized roadblocks on the front tubulin have very different effects on the detachment of the kinesin motor from MT. The small roadblock accelerates the detachment [79,80,81,86], whereas the large roadblock hinders the detachment [83,84,85], which is counterintuitive.

## 6. Velocity Decreases Sensitively with or Depends Insensitively on the Solution Viscosity

**Figure 6:**
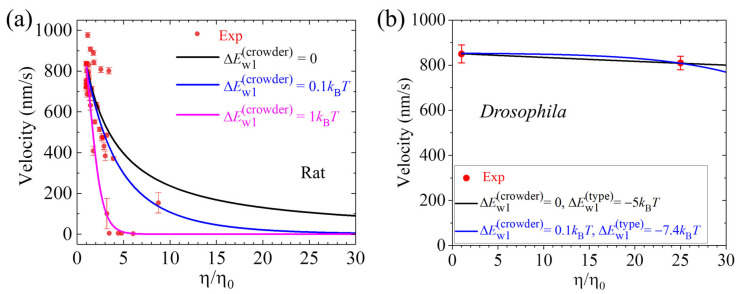
Velocity versus normalized viscosity (*η/η*_0_) for rat and *Drosophila* kinesin motors. *η* is the effective viscosity of the solution with the presence of the crowders and *η*_0_ is the viscosity of the solution with the absence of the crowders. (**a**) Results for the rat kinesin. The experimental results (symbols) are from Sozanski et al. [87]. (**b**) Results for the *Drosophila* kinesin. The experimental results (symbols) are from Nettesheim et al. [88]. In (**a**) and (**b**), the theoretical results (lines) are from Ref. [89]. $\Delta E_{w1}^{\left(\mathrm{crowder}\right)}$ denotes the variation of *E*_w1_ caused by the crowders per increase in the normalized viscosity and $\Delta E_{w1}^{\left(\mathrm{type}\right)}$ represents the variation of *E*_w1_ for the *Drosophila* kinesin relative to that for the rat kinesin.

The influence of the solution viscosity on the velocity of the kinesin motor has been experimentally investigated by two research groups [87,88]. In the experiments by Sozanski et al. [87], the effective solution viscosity was varied by making use of various types, sizes, and/or concentrations of small molecular crowders including polyethylene glycol, dextrans, tetraethylene glycol, sucrose, and bovine serum albumin (BSA). In the experiments of Nettesheim et al. [88], the effective solution viscosity was varied by varying the concentration of BSA. Sozanski et al. [87] observed that, in general, the velocity of the rat kinesin significantly decreases as the effective viscosity increases (Figure 6a). For instance, increasing the viscosity by only about 3 times can decrease the velocity by about 1.6–8 times (Figure 6a). In contrast, Nettesheim et al. [88] observed that increasing the viscosity by 25 times has only a slight influence on the velocity of the *Drosophila* kinesin (Figure 6b).

Together, the experimental results of Sozanski et al. [87] showed that the velocity of the rat kinesin motor decreases sensitively with the increase in the effective viscosity, as shown in Figure 6a. In contrast, the experimental results of Nettesheim et al. [88] showed that the velocity of the *Drosophila* kinesin motor is insensitive to the increase in the effective viscosity, as shown in Figure 6b.

## 7. A Model Can Consistently Explain the Contrasting Experimental Results

In the literature, many models for the chemomechanical coupling of the kinesin motor have been presented, as reviewed elsewhere [56]. In general, those models can be categorized into two types. In one type of the models, it was proposed that the forward stepping of the motor is driven mainly by the NL docking caused by the ATP binding in the leading head [37,48,49,50,51,52], and thus these models were usually termed as the ‘NL-docking’ models [56]. In the other model, it was proposed that the stepping of the motor is achieved mainly by the Brownian ratchet mechanism, with the NL docking playing an assisting role [56,66,67,74,77,78], and thus this model was termed as the ‘Brownian Ratchet’ model [56]. In the NL-docking models, the dependence of the motor’s velocity on the load was explained to arise mainly from the dependence of the motor’s ATPase rate on the load [37,48,49,50,51,52]. Thus, with the NL-docking models, it is difficult to explain the contrasting results about the dependencies of the velocity on the backward load and on the forward load. It is also difficult to explain the decrease in the velocity with the increase in the solution viscosity, because the experimental data indicated that the ATPase rate is independent on the viscosity [87,88]. The counterintuitive experimental results showing that after reaching the large roadblock the pausing time of the motor before dissociation is much longer than after reaching the small roadblock are also difficult to explain.

**Figure 7:**
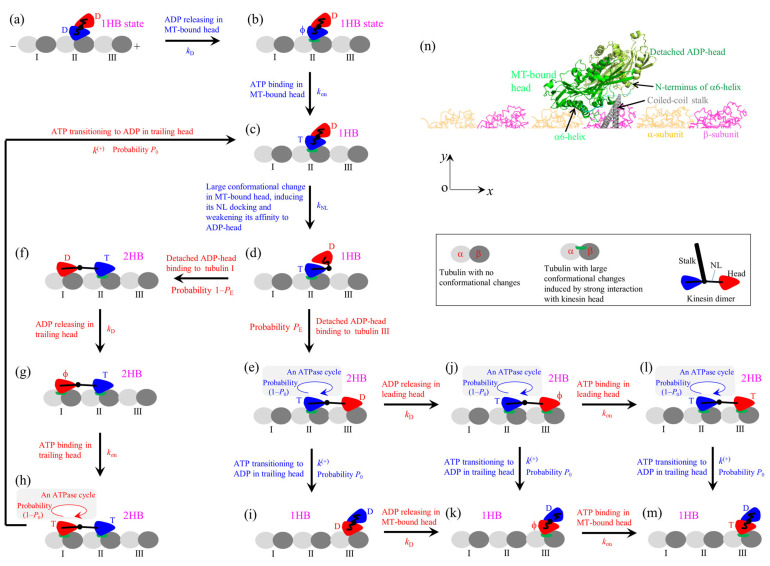
The model for the chemomechanical coupling of the kinesin motor. (**a**–**m**) The pathway at non-saturating ATP concentrations (see Section 7 for detailed descriptions). For clarity, only in the inset the motor’s stalk is drawn. The dissociation of the motor from MT is not drawn. T stands for ATP and ADP.Pi, D stands for ADP, and ϕ stands for nucleotide free. ‘An ATPase cycle’ in (**e**), (**h**), (**j**), and (**l**) represents a chemical cycle of ATP transitioning to ADP, ADP releasing, and ATP binding in the trailing head. (**n**) The position and orientation of the detached ADP-head relative to the MT-bound ϕ-head in the 1HB state, which was determined from AMD simulations [90], with the stalk and two NLs being schematically drawn. The *xyz* coordinates are defined as follows: seen from the motor, the *x*-axis is in the forward direction, the *y*-axis is in the upward direction, and the *z*-axis is in rightward direction.

By contrast, it is noted that the Brownian Ratchet model can consistently explain the contrasting experimental results, as demonstrated before [66,67,74,77,78,89]. The model can be schematically shown in Figure 7 (see Appendix A for main elements, on the basis of which the model is set up). Because the rate of ATP transitioning to ADP in the leading head of its NL in the backward orientation is much lower than that in the trailing head of its NL in the forward orientation, for clarity, ATP transitioning to ADP in the leading head is not shown in Figure 7.

Let us start with one ADP-head binding to tubulin II with weak affinity *E*_w2_ (defined in Appendix A) and the detached ADP-head having a high affinity for the MT-bound head (Figure 7a). The high affinity prevents the detached head from binding to MT. Activated by MT [91], ADP releases from the MT-bound head (Figure 7b). After ATP binding (Figure 7c), a large change in the conformation of the ATP-head, which is associated with the docking of its NL onto the head and the great reduction of its affinity for the detached head, takes place (Figure 7d). The detached head can with a probability *P*_E_ diffuse forward rapidly (with a timescale of 1 μs) and bind to tubulin III with affinity *E*_w2_ (Figure 7e) while with probability 1–*P*_E_ diffuse backward and bind to tubulin I with affinity *E*_w2_ by overcoming the energy barrier resulting from the large change in the conformation of the ATP-head and its NL docking (Figure 7f).

In Figure 7f, ADP releases from the trailing head (Figure 7g), followed by ATP binding (Figure 7h). After ATP transitioning to ADP in the trailing head, driven by the internal force resulting from the NL stretching and by overcoming the weak affinity *E*_w1_ (*E*_w1_ << *E*_w2_, see Appendix A) for the local tubulin I, the ADP-head with probability *P*_0_ can detach and diffuse rapidly (with a timescale of 1 μs) to the intermediate (INT) position (Figure 7c). Accordingly, with probability 1–*P*_0_ the ADP-head cannot detach from or after detachment can rebind to tubulin I, followed by ADP releasing and ATP binding.

In Figure 7e, after ATP transitioning to ADP in the trailing head, with probability *P*_0_ the ADP-head can detach and diffuse rapidly to the INT position (Figure 7i), followed by ADP releasing from the MT-bound head (Figure 7k). In Figure 7e, ADP releases from the leading head (Figure 7j), followed by ATP binding (Figure 7l). In Figure 7j, after ATP transitioning to ADP in the trailing head, with probability *P*_0_ the ADP-head can detach and diffuse rapidly to the INT position (Figure 7k), followed by ATP binding to the ϕ-head (Figure 7m). In Figure 7l, after ATP transitioning to ADP in the trailing head, with probability *P*_0_ the ADP-head can detach and diffuse rapidly to the INT position (Figure 7m). Figure 7m is the same as Figure 7c, except that the motor makes a forward step in Figure 7m.

As the AMD simulation showed, in the 1HB state prior to the occurrence of the large change in the conformation of the MT-bound ATP-head, the detached ADP-head is at the leftward-, forward- and upward-biased position of the MT-bound head (Figure 7n) [90]. In the following, we briefly review how the model can consistently and quantitatively explain the contrasting experimental results. For convenience of writing, unless otherwise pointed out, from now on, the 1HB state refers to the one prior to the occurrence of the large change in the conformation of the MT-bound ATP-head.

### 7.1. Explanation of the Conflicting Experimental Results on ATP Binding in 1HB or 2HB State

The conflicting experimental results on ATP binding in the 1HB or 2HB state are briefly explained as follows [66]. Under no load, we have *P*_E_ ≈ 1 and *P*_0_ ≈ 1. The conflicting experimental results are due to distinct effects of the labeling on the interaction between the two heads in the 1HB state. In the experiment of Isojima et al. [27], the attachment of the particle to the amino acid at the rear position of one head via two polypeptide chains has no interference effect on the interaction between the two heads in the 1HB state, where the detached head is at the forward- and upward-biased position of the MT-bound head (Figure 7n). Thus, based on the pathway (Figure 7), the experimental results by Isojima et al. [27] can be theoretically reproduced (Figure 1a) [66,67]. In the experiment of Wolff et al. [28], the labeling of the 1 nm fluorophore at α6-helix C-terminus of one head also has no interference effect on the interaction between the two heads in the 1HB state. Thus, the experimental results by Wolff et al. [28] can also be theoretically reproduced (Figure 1c) [66]. In the experiment of Mickolajczyk et al. [26], the 30 nm particle that is attached to the center right of one head via a short linker of the contour length of only 2.91 nm can interfere with the interaction between the two heads in the 1HB state. The reduction in the affinity between the two heads can result in the detached ADP-head in the 1HB state of Figure 7k to bind to MT prior to ATP binding to the MT-bound head. Thus, this leads to ATP binding mainly in the 2HB state. With this consideration, the experimental results by Mickolajczyk et al. [26] can be theoretically reproduced [66] (Figure 1b).

### 7.2. Explanation of the Contrasting Experimental Results on Velocity Versus Backward Load

The contrasting experimental results on the velocity versus backward load are briefly explained as follows [74]. Because in the optical trapping assays, the trap is kept stationary using the fixed trap whereas the trap can be movable using the movable trap, it is expected that the fluctuation of the trap relative to the bead for the fixed trap is generally smaller than that for the movable trap, inducing the deviation of the position of the kinesin-bead complex relative to the trap from the equilibrium position for the former to be small than that for the latter. This results in that the force-sensitivity distance for the ADP-head to diffuse from the rear tubulin to the INT position (e.g., the transition from Figure 7e–i) for the fixed trap is larger than that for the movable trap, giving *P*_0_ < 1 for the former and *P*_0_ ≈ 1 for the latter under the backward load. *P*_0_ can be approximately written as $P_{0}=\exp\left(-\beta F\Delta_{1}\right)$, where $\beta^{-1}=k_{B}T$ is the Boltzmann constant times the absolute temperature, *F* is the backward load, and $\Delta_{1}$ is the force-sensitivity distance, with $\Delta_{1}$ ≈ 0 for the movable trap and $\Delta_{1}$ having a non-zero small value ( ≤1 nm) for the fixed trap [74]. *P*_0_ < 1 leads to the velocity decreasing quicker with the backward load than *P*_0_ ≈ 1. Thus, while the velocity versus backward load with *P*_0_ ≈ 1 has the sigmoid form (Figure 2a,b), with *P*_0_ < 1 has the nearly linear form (Figure 2c,d) [74].

### 7.3. Explanation of the Contrasting Experimental Results on Velocity Versus Forward Load

**Figure 8:**
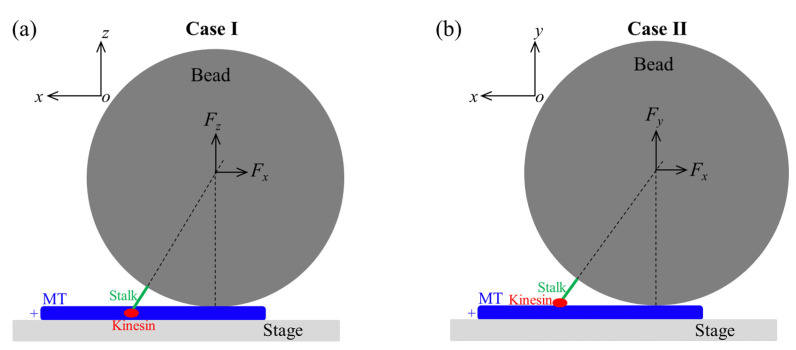
Diagram showing the relative location between the large-sized bead and kinesin motor. (**a**) Case I: the bead on the right side of the motor (viewed in the plus-ended direction). (**b**) Case II: the bead on the upper side of the motor. Seen from the kinesin motor, the coordinates defined here are the same as those in Figure 7, with the *y*-axis being perpendicular to the plane of the paper and outward in (**a**) while the *z*-axis being perpendicular to the plane of the paper and inward in (**b**).

The contrasting experimental results on the velocity of kinesin-1 versus forward load are briefly explained as follows [74]. Under no or forward load, we have *P*_E_ ≈ 1 and *P*_0_ ≈ 1. In the optical trapping assays, MT is fixed to the stage surface and the kinesin with its stalk attached to the large-sized bead is bound to MT. The contrasting experimental results are due to distinct positions of the kinesin on MT. One case (Case I) is that the motor is on the left side of MT (viewed in the plus-ended direction) and the other case (Case II) is that the motor is on the upper side, where the bottom side contacts the stage surface (Figure 8). In the 1HB state, since the detached head is at the forward-biased position of the MT-bound head (Figure 7n), the backward and forward loads on the stalk acts mainly on NLs of the detached and MT-bound heads, respectively. The structural studies demonstrated that the large change of the conformation of the head in ATP state relative to that in ϕ state involves a large-scale rotation of α6 helix relative to α4 helix binding to MT, with α6-helix C-terminus moving leftward for a large distance [17,18,19,20,44,92]. Thus, for Case I (Figure 8a), the forward load on the bead, giving a rightward component (*z*-component) of the load on the stalk that points rightward, can reduce the rate (*k*_NL_) of the leftward movement of α6-helix C-terminus; namely, the large change in the conformation of the MT-bound ATP-head and its NL docking [74]. In contrast, for Case II (Figure 8b) the forward load, giving no rightward component (*z*-component) on the stalk that points upward, has no effect on *k*_NL_ [74]. Therefore, for Case I, the decrease in *k*_NL_ with the forward load results in the decrease in the velocity, whereas for Case II, the velocity is nearly independent on the forward load (Figure 3) [74].

The experimental results that the velocity of the WT human kinesin-1 is nearly independent on the forward load, whereas that of the mutant one with the extended NLs increases with the forward load, are briefly explained as follows [77]. For the mutant kinesin-1, we still have *P*_E_ ≈ 1 under no or forward load. However, due to the NL extension, the internal force between the two heads in the 2HB state becomes nearly zero, leading to *P*_0_ < 1 under no load. Thus, for Case II the velocity increases with the forward load because *P*_0_ increases with the forward load (Figure 4) [77]. Additionally, under no load, *P*_E_ ≈ 1 and *P*_0_ ≈ 1 for the WT kinesin-1 give nearly one ATP consumed per step whereas *P*_E_ ≈ 1 and *P*_0_ < 1 for the mutant kinesin-1 give multiple ATPs consumed per step [77], which are consistent with the experimental results [93,94]. As for the mutant kinesin-1, for the kinesin-2 KIF17 and kinesin-12 KIF15, it is also considered that *P*_0_ < 1 under no load, although *P*_E_ ≈ 1 under no or forward load [78]. Thus, the velocities of KIF17 and KIF15 also increase with the forward load for Case II (Figure 5) [78].

### 7.4. Explanation of the Conflicting Experimental Results on Pausing Time upon Reaching Roadblocks

**Figure 9:**
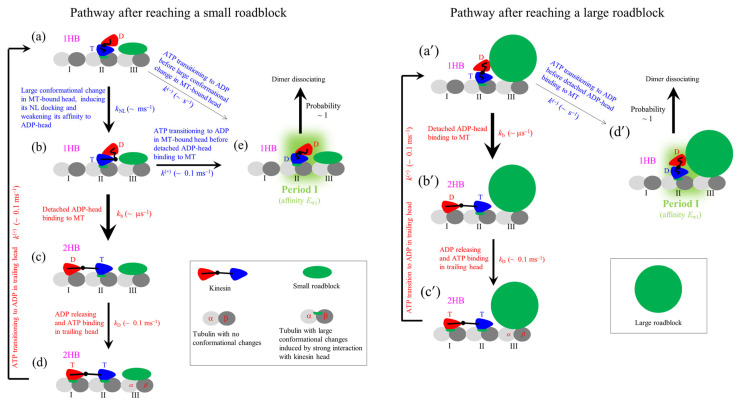
Chemomechanical pathways of the kinesin motor after reaching roadblocks at saturating ATP concentrations. The pathways are derived from the pathway shown in Figure 7. Note that *P*_0_ ≈ 1 under no load. (**a**–**e**) The pathway for the roadblock having a small size. (**a’**–**d’**) The pathway for the roadblock having a large size. The arrow’s thickness is drawn to be roughly proportional to the magnitude of the transition rate. The orders of the transition rates are indicated in the figure, where *k*^(+)^ stands for the rate of ATP transitioning to ADP in the head of its NL in the forward orientation, *k*^(−)^ stands for the rate of ATP transitioning to ADP in the head of its NL not in the forward orientation, *k*_NL_ stands for the rate of the large change in the conformation of the ATP-head together with the docking of its NL and the weakening of its interaction with the detached ADP-head, *k*_b_ stands for the MT-binding rate of the detached ADP-head when its interaction with the MT-bound ATP-head becomes weak, and *k*_D_ stands for the ADP-releasing rate from the MT-bound ADP-head.

The conflicting experimental results on kinesin pausing for a short or long time upon reaching a roadblock are briefly explained as follows. It was shown that with the front tubulin III in Figure 7 being occupied by a small roadblock, the residence time of the kinesin motor on MT is determined mainly by the occurrence probability of the event that ATP transitioning to ADP in the MT-bound head takes place before the detached ADP-head binding to the rear tubulin I [95]. If the event occurs, the period (called Period I) occurs when the MT-bound head has the very weak affinity *E*_w1_ for the local tubulin and the other head is detached from MT, during which the motor can dissociate easily from MT (Figure 9). The conflicting experimental results arise from different-sized roadblocks on the front tubulin having different interference effects on the location of the detached head relative to the MT-bound head in the 1HB state.

The small roadblock has no interference effect on the location of the detached head relative to the MT-bound head in the 1HB state (Figure 9a) [95]. Before NL docking of the MT-bound head (Figure 9a), due to the very low rate of ATP transitioning to ADP, Period I can occur with a low rate (Figure 9, transition from Figure 9a–e). After NL docking of the MT-bound head (Figure 9b), due to the high rate of ATP transitioning to ADP, Period I can occur with a high rate (Figure 9, transition from Figure 9b–e). Hence, the kinesin motor has a short residence time on MT. By contrast, the large roadblock can have an interference effect on the relative location of the detached ADP-head to the MT-bound head in the 1HB state (Figure 9a’), because the detached head is at the forward- and upward-biased position of the MT-bound head. Consequently, in the 1HB state, the detached ADP-head cannot have the high affinity for the MT-bound head (Figure 9a’) and thus the detached ADP-head can bind to the rear tubulin before the large change in the conformation of the MT-bound ATP-head associated with its NL docking (Figure 9b’). Due to the very low rate of ATP transitioning to ADP in the MT-bound head prior to its NL docking, Period I can occur with a low rate (Figure 9, transition from Figure 9a’–d’). Hence, the kinesin motor has a long residence time on MT.

### 7.5. Explanation of the Conflicting Experimental Results on Dependence of Velocity Upon Solution Viscosity

The conflicting experimental results on the velocity versus solution viscosity are briefly explained as follows [89]. The experimental results indicated that the presence of the small crowders in the solution has little influence on the ATPase activity of the kinesin motor [87,88]. Under no load, we have *P*_E_ ≈ 1 in either absence or presence of the crowders. As noted above, we have *P*_0_ ≈ 1 under no load in the absence of the crowders. The presence of the crowders increases the effective viscosity [87,88]. Moreover, the crowders can alter slightly the interaction of the head with MT, with the affinity *E*_w1_ being increased by a few *k*_B_*T*. Either the increase in the viscosity or the increase in the affinity *E*_w1_ can induce *P*_0_ < 1. Thus, the experimental results by Sozanski et al. [87] showing that the velocity of the rat kinesin decreases significantly as the effective viscosity increases can be explained (Figure 6a) [89]. By considering that the affinity *E*_w1_ for the *Drosophila* kinesin is smaller than that for the rat kinesin, the experimental results by Nettesheim et al. [88] showing that increasing the viscosity by 25 times has only a slight influence on the velocity of the *Drosophila* kinesin can also be explained (Figure 6b) [89].

### 7.6. Explanation of Other Experimental Results

The model can also explain well other experimental results about the dynamics of the kinesin motors [55,56,57,58,77,78,96]. For instance, the experimental results about dependencies of the run length and dissociation rate on both backward and forward loads for members of different families such as kinesin-1, kinesin-5 Eg5, kinesin-12 KIF15, kinesin-2 KIF17, kinesin-2 KIF3AB, etc., were explained [78]. The perplexing experimental results about bidirectional motions of some kinesin-5 dimers such as *S. cerevisiae* Cin8 and Kip1 and *S. pombe* Cut7, with the direction depending on the experimental condition albeit with the NL always docking in the forward orientation [97,98,99,100,101], were explained [102]. The puzzling experimental results for orphan kinesin PAKRP2 having a long NL of 32 amino acids in each head, which like kinesin-1 can also move hand-over-hand by hydrolyzing one ATP per step [103], were explained [104]. The experimental results about some families of kinesin dimers such as kinesin-1, kinesin-2, and kinesin-5 moving on MT in the predominant 2HB state under saturating ATP concentrations [17,39,65,105], whereas others such as kinesin-3 and kinesin-13 moving in the predominant 1HB state [106,107,108], were explained [67,109]. The experimental results about the dynamics of motors with various mutations, such as the deletion or mutation of the N-terminus cover strand contributing to the NL docking in kinesin-1 [35], the NL extension in kinesin-1 [37], the NL mutation in kinesin-1 [110], the swapping of NL in kinesin-2 with that in kinesin-1 [111], the joining of the stalk and neck of kinesin-14 Ncd to the head of kinesin-1 [112], the replacement of the head of kinesin-1 with that of Ncd [113], etc., were explained [77]. The perplexing experimental results showing that the transport of cargo by two or multiple kinesin monomers with short truncated stalks connecting to the cargo behaves as efficiently as that by one or multiple kinesin dimers [114,115] were explained using the similar model [116].

## 8. Concluding Remarks and Future Perspectives

Here, a brief review of diverse contrasting experimental results about the dynamics of the kinesin motor is presented. The contrasting experimental results include (1) those on whether ATP binding occurs in the 1HB or 2HB state, (2) those on whether the velocity versus backward load has a sigmoid or linear form, (3) those on whether the velocity decreases with or is nearly independent on or increases with the forward load, (4) those on whether the motor pauses for a short or long time upon reaching a stationary roadblock, and (5) those on whether the velocity decreases sensitively with or is insensitive to the increase in the solution viscosity. Furthermore, a model for the chemomechanical coupling of the kinesin motor (Figure 7) is briefly reviewed, which can consistently explain these contrasting experimental results as well as other experimental results. A consistent explanation of the diverse contrasting experimental results with one model is an essential criterion for determining whether the model is correct or not for the chemomechanical coupling mechanism of the kinesin motor.

A peculiar characteristic for the model of Figure 7 is that for the WT kinesin motor, while under no or low backward loads, approximately one ATP is consumed per forward step; under the medium or large backward loads, multiple ATPs are consumed per forward step. Based on the model of Figure 7, for the rat kinesin motor, the reduced velocity under the increased viscosity and under no load (Figure 6a) is due to the increased futile chemomechanical cycles or increased number of ATPs hydrolyzed per forward step. Based on the model of Figure 7, it is predicted that after reaching a roadblock, multiple ATPs are hydrolyzed before dissociation and, moreover, after reaching the large roadblock, much more ATPs are hydrolyzed than after reaching the small roadblock. To further confirm the validity of the model of Figure 7, it is hoped to test these predictions through future experiments.

## Data Availability

Data sharing is not applicable to this article as no datasets were generated or analyzed during the current study.

## Abbreviations

ADP	adenosine diphosphate
AMD	atomistic molecular dynamics
ATP	adenosine triphosphate
ATPase	adenosine triphosphatase
BSA	bovine serum albumin
ϕ	nucleotide free
INT	intermediate
MT	microtubule
NBP	nucleotide-binding pocket
NL	neck linker
1HB	one-head-bound
2HB	two-heads-bound
WT	wild type

## Appendix A. Main Elements in the Model of Figure 7

The main elements, which are derived from the published structural, biochemical, and AMD simulation studies and on the basis of which the model of Figure 7 is set up, are summarized as follows.

***Interaction between kinesin head and MT***—The head in ϕ and ATP (ATP representing both ATP and ADP.Pi) states shows a strong interaction, whereas in the ADP state shows a weak interaction with MT [12,117,118]. The strong interaction induces large conformational changes, whereas the weak interaction induces little conformational changes of the local tubulin on MT, as shown by cryo-electron microscopic structural studies [21,119] and by AMD simulation studies [120]. The ADP-head shows a much weaker affinity (*E*_w1_) for the tubulin having the large conformational changes than the affinity (*E*_w2_) for the tubulin having little conformational changes, as indicated by AMD simulation studies [120,121]. Thus, it is expected that after ATP transitioning to ADP in the MT-bound head, there exists a short time *t*_r_ (with the timescale of 10 μs) when the ADP-head shows affinity *E*_w1_ for the local tubulin and in time *t*_r_ the local tubulin returns elastically to the normally unchanged form, with the affinity of the ADP-head for the local tubulin transitioning from *E*_w1_ to *E*_w2_ [55,104]. The evidence that kinesin binding can induce the conformational changes of MT, in turn affecting the affinity of kinesin for the MT, is also supported by the fact that the kinesin binding can affect the binding of other kinesins to the MT [119,122,123,124] and particularly the velocity and run time of a kinesin-4 motor can be largely influenced by other distant kinesins on the MT [125], which can be explained by the propagation of the MT conformational changes along the MT [126].

***Nucleotide-dependent conformation of the kinesin head and its interaction with the partner head***—In ADP and ϕ states, the head possesses the conformation, having an open nucleotide-binding pocket (NBP) and an NL incapable of docking onto the head, as indicated by structural and experimental studies [17,18,127,128,129], and with the head showing a large affinity for the partner ADP-head, as indicated by AMD simulation studies [90]. In the ATP state, the NBP closing and a large change in the conformation of the head can take place, so that NL becomes capable of docking onto the head, as indicated by structural and experimental studies [17,18,127,128,129], and the affinity of the head for the partner ADP-head decreases largely, as indicated by AMD simulation studies [90]. NL driven in the backward orientation inhibits the NBP closing and the large change in the conformation of the ATP-head [130,131].

***ATPase activity of kinesin head***—The rate of ATP transitioning to ADP in the head of its NL in the forward orientation is much larger than that of its NL not in the forward orientation, which is supported by the following structural and experimental evidence. The structural studies indicated that NL in the forward orientation clashes with a nucleotide-binding motif (a P-loop subdomain) in the ϕ-orientation, implying that the P-loop subdomain is in the ATP-like orientation [20], which should significantly increase the rate of ATP transitioning to ADP. This is in accordance with the experimental results showing that the mutation or deletion of the NL of the kinesin-1 or kinesin-3 KIF1A head significantly reduces the ATPase rate while it has no effect on the ADP-release rate [20,132], because after ATP binding, NL docks rapidly in the forward orientation. Moreover, the rate of ATP transitioning to ADP in the head is independent on the force acting on its NL. This is consistent with the experimental evidence that extending NLs has little effect on the ATPase rate of the kinesin-1 dimer [93] and the ATPase rate of the truncated kinesin-1 monomer is nearly equal to that of the dimer [24,133,134], because extending NLs largely reduces the stretching force on the NLs of the dimer in the 2HB state [37,55,135] and no stretching force is present on the NL of the monomer.
